# Treatment of Bubo by Excision

**Published:** 1893-06

**Authors:** F. S. Watson

**Affiliations:** Boston


### Selections and Abstracts.

### TREATMENT OF BUBO BY EXCISION.

BY DR. F. S. WATSON, BOSTON.

Dr. Watson has treated twenty cases of bubo by excision, followed by attempt to secure union of the wound by first intention. The buboes were of syphilitic, chancroidal, gonorrhœal, tuberculous and traumatic nature, and in most of the cases the conditions were extremely unfavorable to primary union. In all the operations the following rules were carefully observed:

1. To remove thoroughly all diseased tissue and to leave, as far as possible, a perfectly healthy surface in every part of the wound. (To secure this it is always necessary to carry the dissection down to the fascia covering the abdominal muscles; sometimes to expose the femoral vessels, and generally the external inguinal ring).

2. To excise such portions of the skin as threatened to become necrotic or had already become so.

3. To curette the under surface of the skin flaps.

4. To thoroughly swab the whole wound with dry sterilized gauze-sponges wet with a solution of corrosive sublimate 1-4,000.

Two precautions should be especially observed: First, to guard against wounding the femoral vessels and the spermatic cord, and to proceed with especial caution when dissecting about the inguinal canal, lest an unsuspected hernia be wounded. Second, the divided lymph vessels should be tied as far as possible, to prevent exudation into the wound cavity.

In cases in which the skin was not at all, or moderately inflamed, one of two incisions was employed: 1. A crescentic cut carried well below the area of inflamed skin through the healthy skin. A large flap was then dissected up, exposing the diseased glands, which after their removal was turned down and sutured on the line of the first incision. In some cases drains of sterilized silk were inserted through an opening below the line of incision. 2. A long incision parallel with Poupart's ligament, through the skin across the middle of the swelling. The skin was dissected up, the glands exposed, and the wound sutured without drainage. The first incision was preferred. In cases in which the skin covering the bubo had broken down, the last incision was employed, being modified by dividing it towards its middle so as to form an ellipse, which included the area of necrotic skin and so removed it. In the author's cases enough sound skin was left to permit approximation of the wound margins. Secondary suture was resorted to in some cases, the sutures being introduced at the time of operation, but the wound left open and packed with iodoform gauze for twenty-four or more hours, when it was closed. This method has the advantage of allowing free evacuation of fluids after the operation.

Excision of buboes in the above described manner, is claimed by the author to materially abridge the period of healing of buboes. In cases in which the wounds healed by primary union the average duration was sixteen days; in those healing by granulation it was thirty-four days.—*Journ. of Cutan. and Genito-Urin. Diseases,*

---

SCARLET FEVER IN ADULTS.—GIMMEL (*Deutsches Archiv. fur klinische Medicin*, Vol. LI, Part 1) reports 1818 cases of scarlet fever which occurred in Zurich in eight years; 274 of these cases were in adults, or 15 8-100 per cent. of the whole number. He concludes his article as follows: (1) Adults are somewhat less disposed to scarlet fever than children. (2) The longer the time which has elapsed without an epidemic of scarlet fever, the greater the number of adults seized in the epidemic when it occurs. (3) The course is less severe in adults than in children. (4) Nephritis is less common in adults than in children, and is not so severe. (5) Articular rheumatism is a more frequent complication in adults than in children. (6) Scarlet fever, conveyed by wounds, occurred in the observer's cases only in adults.—*Ex.*

